# NEC in Twin Pregnancies: Incidence and Outcomes

**Published:** 2014-10-20

**Authors:** Sathyaprasad C Burjonrappa, Brian Shea, Diya Goorah

**Affiliations:** Children’s Hospital of New Jersey, USA

**Keywords:** NEC, Twin, Pregnancy

## Abstract

Background: Necrotizing Enterocolitis (NEC) is the most common gastrointestinal emergency in neonates. Previously established risk factors for the development of NEC include prematurity and low birth weight. However, it is not clear to date as to whether the etiology of NEC is due to host, environmental, or yet other unknown factors. We analyzed the differences in incidence of NEC in twin pregnancies to further clarify its etio-pathogenesis.

Methods: After IRB approval, a retrospective search of the medical records of the Department of Pediatric Surgery was done to identify all the neonates treated for surgical NEC from 2006-2013. Patients that had been treated for NEC elsewhere and subsequently transferred in to our facility were excluded. The medical records of the resulting 45 patients were then analyzed for demographics, antenatal screening, risk factors, treatment (medical and surgical), and outcomes. The resulting data was then analyzed using relative risk calculations and standard statistical tests.

Results: Of the 45 patients who developed surgical NEC, 9 neonates (20%) were born of a twin pregnancy. There were no cases in which both twin A and twin B developed NEC. NEC in twin pregnancy neonates showed a female preponderance (p less than 0.0001) and developed universally in the first born of the twins. Birth weight, time of onset of NEC, hospital stay and mortality were similar between twin and non-twin NEC. There was an average lead-time of three weeks to development of NEC in both singletons and twin pregnancies.

Conclusion: There is a remarkable higher incidence of NEC amongst twins. Abnormal colonization of the gastrointestinal tract appears to be an immediate postpartum event. NEC in twin pregnancy does not appear to have a deleterious outcome compared to NEC in singleton pregnancy.

## INTRODUCTION

Necrotizing Enterocolitis (NEC) is the most common gastrointestinal emergency in neonates occurring in 1 to 3 per 100 live births. The incidence is higher in premature and very low birth weight (VLBW) infants (less than 1500g), occurring in approximately 10% of these newborns.[1] Twin studies have played an important role in defining the role of nature and nurture in determining the predisposition to disease.[2] While twin studies have been conducted on NEC in the past, there has been a paradigm shift in the understanding of the etiopathogenesis and management of this condition over the past 10 years.[3,4] We looked at NEC outcomes in light of the new therapeutic strategies available in its management and also aimed to understand its pathophysiology in the setting of twin pregnancies. 

Our secondary objective was to evaluate the role of the microbiome in twin pregnancies in causing NEC. The gut microbiome develops transiently during the birthing process with subsequent rapid colonization from the immediate environment.[5] A recent study found that in a variety of exposures, preterm twins shared more similar gut microbiomes compared to preterm infants who were non- genetically related.[6] Albeit controversial, studies aiming to alter the gut microbiome have become the focus of researchers and there is a growing body of evidence that suggests a role for probiotics in the prevention of NEC.[7,8] We hypothesized that in the standardized care offered by the modern Neonatal Intensive Care Units (NICU), the development of the intestinal microbiome should be no different amongst a set of twins and hence if an imbalance in the microbiome were to be a major cause of NEC, both twins should have an equal predisposition to and incidence of NEC.

## MATERIALS AND METHODS

A retrospective search of the medical records of the Department of Pediatric Surgery at a tertiary referral center was used to identify all neonates treated for surgical NEC from 2006-2013 based on ICD-9 diagnosis codes. Surgical NEC by definition implied that the neonate needed peritoneal drain placement or laparotomy. Patients transferred to our facility for management of surgical NEC after birth at outside institutions and those solely treated medically were excluded from further analysis. This was done to ensure that the study subset included only neonates born at a single facility and in a standardized environment. For the study purposes, it was assumed that a relatively homogenous population (with exposure to the same external environment) would better serve to answer the question as to whether the development of the gut microbiome influenced the incidence of NEC in twin pregnancies. The obstetric operating theaters, delivery rooms, and the NICU being on the same floor, at the facility, the newborns needing admission to the unit were not transported over significant distances. This reduced exposure to a non-standardized environment. In preterm labor, the obstetrician based on the maternal and fetal condition decided upon the mode of delivery. Expressed breast milk was encouraged as the best nutrition option for the preterm newborn and every effort was made to start and advance feeds by gavage at a slow rate unless otherwise contraindicated. Strict unit policy with regards to hand washing for medical and nursing staff and isolation policies for neonates with communicable infections or sepsis aimed to reduce the incidence of nosocomial infections. Surgical NEC was managed by drain placement in the micro-premies and laparotomy was considered for neonates greater than 1500g or for those who failed to improve after drain placement. The medical records of the resulting 45 patients were then analyzed retrospectively by chart review. Prenatal factors evaluated included race, presence or absence of antenatal care, gestational age, mode of delivery, and twin or singleton pregnancy. Post natal factors examined included demographics, birth weight, gestational age, birth complications, congenital abnormalities, timing of initiation of feeds, type of feeds, need for ventilator support, timing of onset of NEC symptoms, timing of surgical intervention, indication for surgery, and surgical procedure. 

Twin pregnancies were evaluated for development of NEC in one or both twins. The factors that could have contributed to differential development of NEC in twin pregnancies were evaluated based on known antenatal and postnatal risk factors for the condition. The primary outcome measured was concordance for NEC development in sets of twin pregnancies. Secondary data points evaluated included 90-day post-operative mortality, length of hospital stay, and difference in NEC outcome between singleton and twin pregnancies. The data was analyzed using relative risk calculations and standard statistical tests. Chi-square test was used to test the statistical significance of differences in categorical data and the student t-test was used to evaluate difference in continuous variables.

## RESULTS

There were 45 cases of surgical NEC during the study period of which 9 (20%) were born of a twin pregnancy. The clinical characteristics and outcomes of the study subset are summarized in Table 1. Seven of the twin pregnancies were dizygotic. One of the conceptions was actually a triplet pregnancy where selective reduction of an anencephalic fetus was performed. Two of the twin pregnancies were complicated by twin-twin transfusion with fetal death of one of the twin pregnancies. Eight of the nine twin conceptions went on to delivery of live twins. There were no cases in which both twins developed NEC. NEC in twin pregnancy neonates showed a female preponderance (78%; p less than 0.0001) and occurred universally in the first born of the twins. The ethnic distribution of the newborn was no different between singletons and twin pregnancies however 89% of all twin deliveries were by c-section as compared to 61% of singleton deliveries (p less than 0.0001). There was no difference in the APGAR scores between singleton and twin pregnancies at 1 (p=0.17) and 5 minutes (p=0.613). A majority of the infants in both the twin and singleton NEC groups had formula feeds at initiation of oral feeding as compared to breast milk. The average birth weight for twins with NEC was 850.5g compared to 1061.72g in the non-twins (p=0.411, NS). A higher proportion (89%) of the twin NEC population had been intubated prior to the surgical consultation for NEC. (p=0.0008). The age of onset of NEC was similar between the twin and non-twin pregnancies (p=0.8996, NS). There was no difference in the utilization of drain versus laparotomy as the initial surgical option between the two groups (p=0.453). A majority of infants in both groups, 7/8 in the twin NEC group and 31/36 in the singleton NEC group, ended up having a laparotomy. The overall survival was similar between groups (p=0.8865). 

**Figure:**
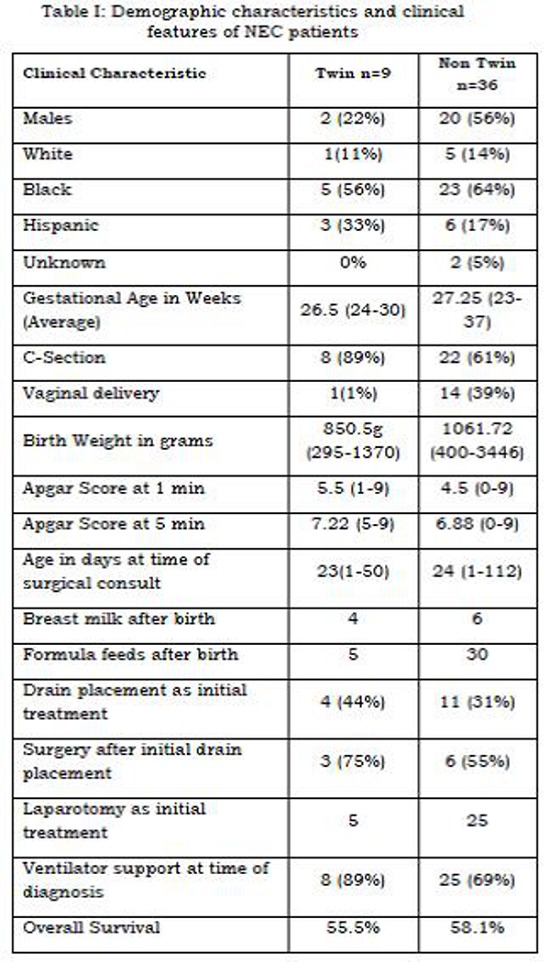
Table I: Demographic characteristics and clinical features of NEC patients

## DISCUSSION

Twin studies allow us to compare the effects of genetics and environmental factors in the pathophysiology of disease processes. A recent study analyzed the fecal microbiome development in healthy preterm infants and those who subsequently developed NEC. While the healthy subgroup demonstrated a temporal pattern in their fecal microbiome development that resembled full term breast fed infants, the ones that developed NEC diverged from the healthy controls around three weeks prior to the onset of the disease process.[9, 10] The median age of neonates developing NEC in this study was around three weeks in both the singleton and twin pregnancies. Extrapolating the microbiome development study findings to our population, it would seem logical to conclude that that the process of abnormal temporal colonization of the gastro-intestinal tract commences during the birthing process or very early thereafter in those patients who develop NEC. Based on the lead-time to development of surgical NEC that was noted in this study, we believe that subtle differences in the initial insult have significant consequences over the three-week period when the pathology manifests itself clinically. 

Antenatal stressors are considered to be important causative factors of NEC.[11] The incidence of cesarean section for twin pregnancies was significantly higher than those for singletons in this study. This finding more likely reflects a bias in the current obstetric practice to perform cesarean sections in twin pregnancies at our facility. A corollary to this observation is the far higher proportion of vaginally delivered singletons that developed NEC. It is tempting to hypothesize that the discordance in twin NEC pregnancies noted in this study may be due to the protection of the twin pregnancy against the significant stress of the vaginal birthing process by the higher cesarean section rates. Why this would protect one but not the other twin is still debatable and may have something to do with the release of maternal and fetal hormones. Both oxytocin and epinephrine, the principal hormones of childbirth, can cause significant vasospasm. The maternal and fetal hormonal response to the birthing process and its influence on the integrity of the intestinal lining bears further study. The preponderance of NEC in the first born of the two twins also suggests that hormonal influences may have a role in the development of NEC. The occurrence of NEC in both sets of twins in a pregnancy complicated by the twin-twin transfusion syndrome (TTS) has been reported.[12] In our study, there was a fetal demise that complicated one of the two TTS pregnancies. The other TTS pregnancy showed discordance in the occurrence of NEC between the twins. The vascular steal in TTS may predispose the donor twin to NEC. Chorio-amnionitis and group B streptococcus infection was not a significant factor in the development of NEC with a very small proportion of singletons and twin pregnancy in this study being complicated by prenatal infection. Prenatal vascular and hormonal influences on the intestinal membrane permeability and its resistance to pathogens appear to be an important factor in the development of NEC.

The association between blood transfusions and cytokine response has received a lot of attention in NEC studies.[13] The significant cytokine release seen in full-blown NEC is more likely a secondary response to the invasive and pathological microbial profile in the GI-tract rather than being the primary initiator of the pathological process. While the immune system in dizygotic twins are not exactly the same, there are many shared genes, and hence the discordance of the disease process in this twin study argues against the immune system as being the primary initiator of the disease process. Interestingly, this discordance in the development of NEC has been noted in other twin studies too.[14] 

NEC has a broad spectrum in its clinical presentation. It can be mild and non-progressive, and can be effectively managed by bowel rest, antibiotics, intravenous nutrition, and electrolyte repletion. However, approximately 50% of patients may follow a more severe course, necessitating urgent surgical intervention.[15] Surgical intervention can take the form of exploratory laparotomy or peritoneal drainage. A recent study showed that about one third of all neonates with surgical NEC have peritoneal drainage as the initial modality of treatment and in this group more than 50% end up having a definitive laparotomy.[16] In this study we did not observe any difference in the use of peritoneal drainage or the need for subsequent laparotomy after initial drainage in twin and singleton NEC cases. However, a larger proportion of singleton NEC neonates underwent laparotomy as the initial intervention (70% vs 55%). Although we did not demonstrate a statistically significant difference in weight between the two groups, the singletons had higher birth weights than twin neonates. Further, the twin NEC subgroup had a significantly higher incidence of intubated patients at the time of surgical consultation suggestive of a greater physiological insult and may have been the contributing factor to the lower incidence of laparotomy as the initial procedure. The incidence of twinning in pregnancy is around 3% and about 12-15% of all preterm births are due to twin pregnancies and hence it would be expected that the incidence of NEC would be higher as well in twin pregnancies.[14, 17] The incidence of twinning in this study was 20% and confirms our suspicion that pre-term twin deliveries do have a higher incidence of NEC than singleton preterm delivery. 

There was the female preponderance of surgical NEC in twin pregnancies as compared to singletons. Furthermore of the eight cases, where both twins were born alive, there was not a single incidence of synchronous or metachronous onset of NEC in the other twin. Further, the twin that developed NEC was the first born in all instances. This might suggests that hormonal influences that prime the mother and fetus at delivery effect the first infant more than the second born. In this study the survival of twin neonates who develop NEC was no different than singletons that develop NEC.

Limitations to the study include a small sample size and a large proportion of dizygotic twins. Examining a larger database with a higher proportion of monozygotic twins may improve the understanding of NEC development in view of the recent developments in understanding its pathophysiology. NEC is a devastating disease, and there is increasing interest in its pathogenesis. Few studies however, have examined NEC in twin pregnancies. The findings of our study demonstrate the need for further research into the pathophysiology of NEC in twin pregnancies.

## Footnotes

**Source of Support:** Nil

**Conflict of Interest:** None

